# Age, Race, Sex and Cardiorespiratory Fitness: Implications for Prevention and Management of Cardiometabolic Disease in Individuals with Diabetes Mellitus

**DOI:** 10.31083/j.rcm2507263

**Published:** 2024-07-11

**Authors:** Eric Nylén

**Affiliations:** ^1^Veterans Affairs Medical Center, Washington, D.C. 20422, USA; ^2^George Washington University School of Medicine, Washington, D.C. 20037, USA

**Keywords:** cardiorespiratory fitness, diabetes, vital sign, age, race, sex

## Abstract

Physical inactivity and poor cardiorespiratory fitness (CRF) are strongly associated with type 2 diabetes (DM2) and all-cause and cardiovascular morbidity and mortality. Incorporating physical activity promotion in the management of DM2 has been a pivotal approach modulating the underlying pathophysiology of DM2 of increased insulin resistance, endothelial dysfunction, and abnormal mitochondrial function. Although CRF is considered a modifiable risk factor, certain immutable aspects such as age, race, and gender impact CRF status and is the focus of this review. Results show that diabetes has often been considered a disease of premature aging manifested by early onset of macro and microvascular deterioration with underlying negative impact on CRF and influencing next generation. Certain races such as Native Americans and African Americans show reduced baseline CRF and decreased gain in CRF in randomized trials. Moreover, multiple biological gender differences translate to lower baseline CRF and muted responsivity to exercise in women with increased morbidity and mortality. Although factors such as age, race, and sex may not have major impacts on CRF their influence should be considered with the aim of optimizing precision medicine.

## 1. Introduction

**Fig. 1.**
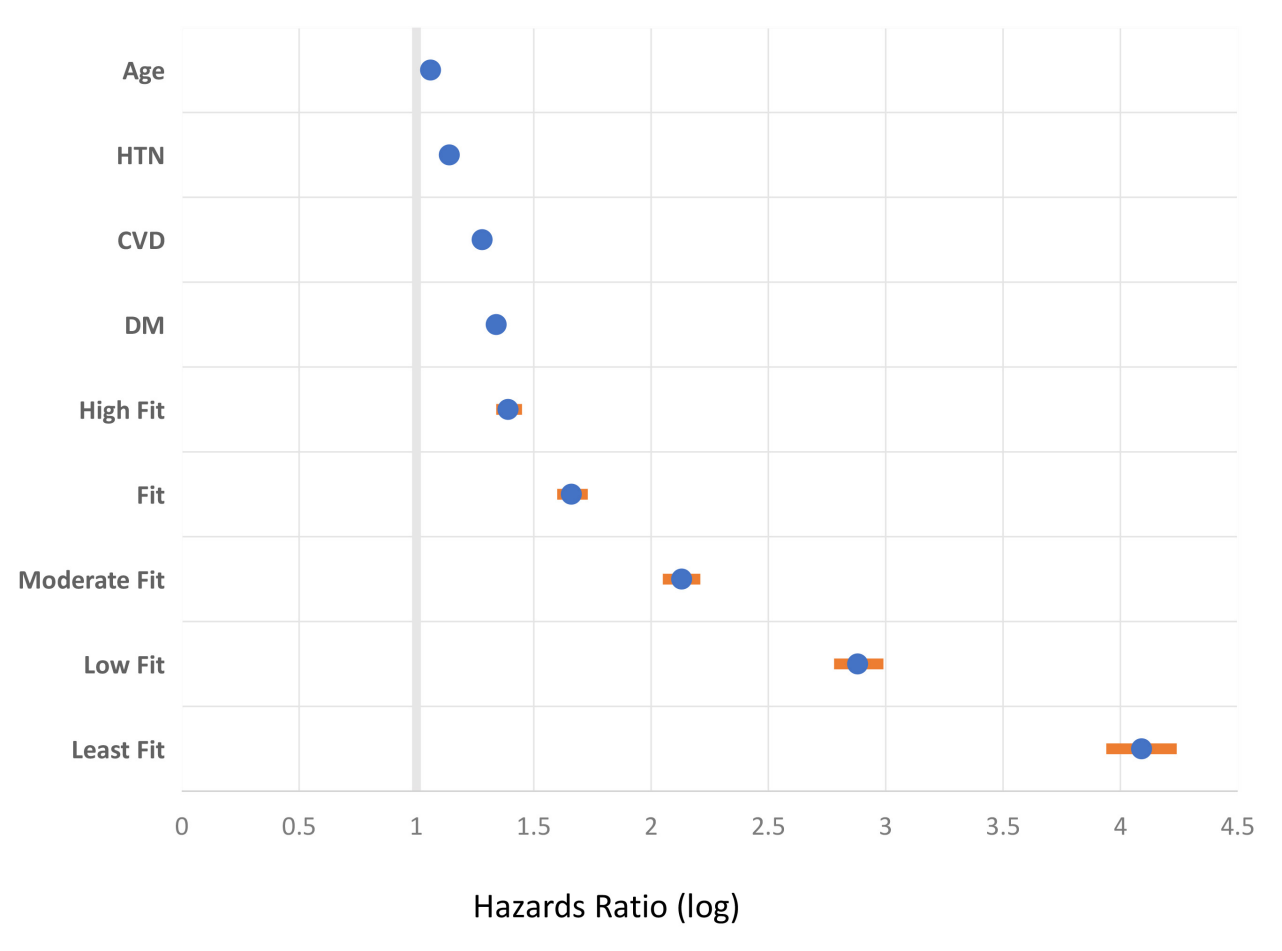
**Cardiorespiratory fitness (CRF) as a superior vital sign compared to selected comorbid risk factors.** The figure shows relative all-cause mortality risk associated with select clinical characteristics among 750,302 US. Veterans that were of multiracial origin, with a wide age spectrum, and of both genders. The figure is a Forest plot of hazards ratio and 95% confidence interval (multivariable fully adjusted Cox regression analysis) (orange line). The CRF was assessed objectively using a standardized exercise tolerance test (Bruce protocol). The extremely fit CRF category was the referent (98th percentile group). Figure is modified from reference [2]. HTN, hypertension; CVD, cardiovascular disease; DM, diabetes. All values are *p* < 0.001.

It is estimated that 11.6 % of the US population have diabetes, dominated by type 2 diabetes (DM2), while another 38% have prediabetes [1]. Among those with diabetes, atherosclerotic cardiovascular disease (CVD) is the leading cause of morbidity and mortality and the cause of disability and reduced quality of life. Lifestyle exercise promotion has been a cornerstone in diabetes management for almost as long as the use of insulin and it has been shown that measured cardiorespiratory fitness (CRF) among diabetes subjects is the strongest predictor of mortality. More recently, a large multiracial epidemiological study reported an inverse, independent, and graded association between CRF and all-cause mortality. More importantly, CRF prognosticated mortality better than any of the traditional risk factors, regardless of age, race, or sex [2] reinforcing the importance of this multi-organ integrative functional metric as a vital sign of health (Fig. 1) [2, 3]. In general, physical activity and CRF levels of subjects with DM2 are substantially lower than those without DM2, and low CRF independently predicts increased risk of morbidity and mortality. Lower CRF is also evident in DM2 patients without CVD and it remains relatively low even after physical activity status is improved [4]. Although the mechanisms involved in this deteriorated baseline CRF are not completely understood, evidence supports that the aerobic pathways maybe compromised including mitochondrial dysfunction [5]. Additional diabetogenic factors such as insulin resistance, vascular, and cardiac dysfunction impact CRF status [6, 7]. Conversely, improvements in CRF lead to more favorable health outcomes in most studies. For example, in the Look AHEAD study, a large randomized controlled trial (RCT) of intensive lifestyle promotion in those with DM2, subjects that increased measured CRF by 2 metabolic equivalents (METs) or more had fewer CVD events [8]. Although CRF is considered a modifying risk factor for CVD, certain attributes such as genetics significantly influence responsivity to exercise [9]. Nevertheless, as reviewed in this article, aging, race (ethnicity), and sex influence CRF status in the diabetic population.

## 2. Age and CRF

Aging in general results in impaired physical function and disabling mobility due to decreased muscle mass and strength characterized by loss of fast-twitch type II myofibers. Diabetes is associated with premature aging characterized by accelerated deterioration of skeletal muscle mass and function [10]. The contributary factors include insulin resistance and hyperglycemia, advanced glycation end-products, increased proinflammation and oxidative stress, and mitochondrial dysfunction. Telomere shortening, a hallmark biomarker of biological aging, is notable in diabetes and telomere length maintenance is responsive to exercise [11]. Multiple studies have shown that that individuals with low CRF have increased risk of developing new onset diabetes (NODM), and a direct causality was suggested by Mendelian Randomization analysis [12]. The CRF-NODM association is linear, inverse, and independent of comorbidities [13]. In a large meta-analysis, the risk of developing NODM was 8% lower for each 1-MET increase in exercise capacity [14].

Exercise is thus a very promising approach to intervene in the aging process as CRF is causal and partly mediated by the effect of fitness on insulin resistance [12, 15, 16]. However, aging overall impacts CRF in a non-linear fashion as shown in the Aerobics Center Longitudinal Study [17]. In this cohort, the age-related decline in CRF for diabetic patients ages 45–60 years with relatively poor CRF was accelerated while the decline for those with relatively high baseline CRF was similar to the decline noted in non-diabetic controls [17]. A comprehensive study of mitochondrial function in otherwise healthy men and women between ages 22 and 80 years found no evidence that aging has a negative impact on mitochondrial function [18]. In contrast, findings support that DM2 as the underlying etiology of mitochondrial dysfunction [19]. Studies also support that DM2 patients and their non-DM2 offspring exhibit insulin resistance and mitochondrial dysfunction [20]. More advanced DM2 is frequently associated with neuropathy and a serious facet involve cardiac autonomic dysfunction which worsens with age which is evident both at rest and following exercise [21].

## 3. Race (Ethnicity) and CRF

Across the globe, at least a quarter of adults are considered physically inactive contributing to low CRF and greatly causative to the burden of non-communicable diseases such as diabetes. As CRF varies among populations, physical inactivity was reported to be the highest in women in Latin America and the Caribbean, followed by those in South Asia and Western countries (all nearly at 40% inactivity) while men in Oceania had the lowest amount of inactivity [22]. Indeed, in the last 20 years there has been a significant general decline in measured CRF with almost a doubling of those in the low CRF category [23].

In the US, the lifetime risk of NODM, and its associated low CRF, is higher for Hispanic (not a race but an ethnic designation) and African Americans compared to Caucasians, especially among women [24, 25]. Overall, African Americans have lower CRF compared Caucasians. Although all-cause mortality among both African American and Caucasian men with DM2 correlates with CRF, this association appears to be stronger for Caucasians. In at least one study, 1-MET increase in CRF resulted in 19% lower risk of all-cause mortality in Caucasians and 14% in African Americans [26]. However, in the HERITAGE family study, race had the least amount of influence on mortality risk compared to sex and age [27]. Interestingly, African Americans with overall lower CRF also have a greater percentage of type II skeletal muscle fibers characterized by reduced oxidative capacity and capillary density [28]. In the Look AHEAD RCT of intensive lifestyle intervention in DM2 in a racially/ethnically diverse cohort there was no impact on CVD events unless CRF improved [8, 29]. However, this inverse association was evident only in Caucasians, while only modest CRF changes were noted in African Americans and Native Americans with no association to CVD [30, 31]. Data from cardiac rehabilitation programs also show that African American subjects with diabetes is the subgroup with the least gains in CRF improvement [32].

## 4. Sex Differences and CRF

The fundamental genetics and biology of men and women contributes to differences in expression of diabetes and in CRF status. Men are typically diagnosed with diabetes at an earlier age and at a lower body mass index (BMI) than women. Obesity, a major risk factor for diabetes and impacting CRF, is more common in women than men especially after age 45 [33]. Although there is significant sexual dimorphism between men and women with respect to hormonal levels, relatively high testosterone levels in women and low levels in men are associated with a higher risk of NODM. Metabolically, there are sex differences in substrate storage of lipids and utilization. Lower rate of fat oxidation and an earlier shift to using carbohydrate as the dominant fuel have been reported in men compared to women. This variation in fat oxidation during exercise remains largely unexplained by differences in body fatness and CRF, suggesting that circulating estrogen may play a role [34]. It is unclear why women have worse insulin resistance relative to men, and how DM2 status and duration may impact these sex differences.

Interestingly the adverse impact of hyperglycemia may be related to a mitigated response of Glucagon-like-peptide-1 (GLP-1) to exercise [35].

Women generally have a lower CRF thought to be due to their higher adiposity and smaller heart size and lower stroke volume [36]. Additional causes include lesser physical activity in women across all age groups and less engagement in leisure time physical activity and moderate to vigorous physical activity [37, 38]. Women also display functional limitations and worse control of diabetes, hypertension, and BMI [39, 40, 41]. These attributes in women with diabetes translates into a higher burden of CVD and heart failure correlating to the degree of adiposity and other CVD risk factors [42, 43]. The so-called diabetic cardiomyopathy is more often seen in women who lose the protective effect of estrogen to cardiomyopathy [44]. In the Look AHEAD RCT, women in the lifestyle group achieved a lower change in CRF compared to men [30]. In a case-control study, the reduced CRF was attributed to low left ventricular volume and sedentary behavior, the latter behavior more prominent among females [45].

## 5. Future Directions

Significant progress is being accomplished in interdisciplinary lifestyle sciences. Prior studies revealed that a durable weight loss of >5% was required for cardio-metabolic benefits, a weight loss most often not achieved [46]. However, novel more potent pharmacotherapy is now evolving rapidly (e.g., GLP-1 analogs) with results matching the weight loss achieved with bariatric surgery nearing 20%. In this new era of significant weight loss and metabolic management, emerging interactions of CRF with pharmacotherapy needs monitoring [47].

Parallel with advances in pharmacotherapy for weight loss has been technologies addressing the motive forces underlying the salutary CRF impact in healthy adults and children such as that taken by The Molecular Transducers of Physical Activity Consortium sponsoring the creation of a molecular map of exercise response using genomic/epigenomic, proteomic/post-translational, transcriptomic, metabolic/metabolomic, and lipidomic assays [48, 49]. By understanding the factors that contribute to exercise response and its well-known variability it will enhance personalized exercise prescription. In addition to charting changes in healthy humans, studies in comorbid individuals with diabetes show significant changes occurring in the heart undergoing enhanced CRF at the metabolome, proteome and transcriptome level [50, 51, 52].

## 6. Conclusions

Exercise has aptly been viewed as a “polypill” with the enhanced CRF having potent impact on mitigating a wide spectrum of non-communicable chronic disorders [53]. Poor CRF is an important vital sign (Fig. 1) [2] and of particular concern for subjects with diabetes. As reviewed, diabetogenic issues such as prematurely aged mitochondria, muscle fiber type differences between races, and the poor CRF response particular to women contributes to the overall compromised CRF baseline and CVD vulnerability. However, it should be realized that the degree of impact of age, race, and/or sex on CRF is relatively minor when compared to the influence of hereditability and a sedentary lifestyle [54]. Moreover, many external factors have to be accounted for, such as the role of air pollution and the inaccuracy of BMI as a measure of obesity, that may also influence CRF itself and its interpretation [55, 56]. Nevertheless, awareness of how CRF is impacted by age, race, and/or sex could improve therapeutic calibration to reach optimal lifestyle promotion and CVD prevention [57].
